# Evaluation of potential complication of interstitial lung disease with abemaciclib and palbociclib treatments

**DOI:** 10.1002/cnr2.1402

**Published:** 2021-05-03

**Authors:** Hideki Nawa, Takahiro Niimura, Kenta Yagi, Mitsuhiro Goda, Yoshito Zamami, Keisuke Ishizawa

**Affiliations:** ^1^ Department of Pharmacy, Faculty of Pharmacy Shujitsu University Okayama Japan; ^2^ Department of Clinical Pharmacology and Therapeutics Tokushima University Graduate School of Biomedical Sciences Tokushima Japan; ^3^ Clinical Research Center for Developmental Therapeutics Tokushima University Hospital Tokushima Japan; ^4^ Department of Pharmacy Tokushima University Hospital Tokushima Japan

**Keywords:** cyclin‐dependent kinase, database, interstitial lung disease

## Abstract

**Background:**

Various cyclin‐dependent kinase 4/6 (CDK4/6) inhibitors have demonstrated promising anti‐tumor effects. The Japanese Ministry of Health, Labour and Welfare has issued a warning about interstitial lung diseases as an adverse effect of CDK4/6 inhibitors. However, a large‐scale evaluation of potential complications has not been conducted to date, and the occurrence of these adverse effects is unclear.

**Aim:**

The aim of this study was to evaluate the clinical incidence of interstitial lung disease caused by two CDK4/6 inhibitors, abemaciclib and palbociclib, and assess the relationship between each drug and interstitial lung disease.

**Methods and results:**

We evaluated the relationship between the CDK4/6 inhibitors (abemaciclib and palbociclib) and interstitial lung disease in clinical practice using data from the Japanese Adverse Drug Event Report (JADER) database and FDA Adverse Event Reporting System (FAERS) to detect adverse event signals with reported odds ratios (RORs). Furthermore, we performed an adverse event‐time analysis for each drug using data from the JADER database to examine the time of onset of the adverse events.

The analysis of the reports in the JADER database showed that the lower limit of the 95% confidence interval (CI) of ROR for abemaciclib was >1 regardless of age, and a signal was detected. Interstitial lung disease associated with abemaciclib and palbociclib use has been reported, with an average onset period from treatment initiation [median (25th‐75th quartile)] of 65.1 [56.0 days (25.3‐98.3 days)] and 53.1 days [38.0 days (10.8‐76.0 days)], respectively. The analysis of the reports in the FAERS showed that the lower limit of the 95% CI of the ROR for the two drugs was >1, and a signal was detected.

**Conclusion:**

Treatment with abemaciclib and palbociclib is associated with a potential complication of interstitial lung disease, regardless of age.

## 1 INTRODUCTION

In recent years, there has been a rapid increase in various anticancer agents for the treatment of patients with breast cancer. Hormonal and combination therapies are widely used to treat breast cancer. In addition, molecularly targeted drugs have shown promising results in several large‐scale trials.^1^, ^2^ Various approaches have been used in clinical practice to improve the therapeutic efficacy of these drugs, including categorizing patients based on the expression of estrogen receptors and human epidermal growth factor receptor type 2 (HER2).^3^

Cyclin‐dependent kinases (CDKs) such as CDK4 and CDK6 disrupt the control of cell growth and cause cells to multiply in an unregulated manner. Anticancer agents can inhibit the CDKs involved in cell cycle control by inhibiting the activity of the CDK4/6 complex with cyclin D, thereby arresting the progression of the cell cycle and exerting an antitumor effect. Palbociclib is the first CDK4/6 inhibitor with a high selectivity for CDK4 and CDK6. It has been demonstrated to be efficacious in combination with endocrine therapy (eg, anti‐estrogen and aromatase inhibitors) against estrogen receptor (ER)‐positive (ER+) and HER2‐negative (HER2−) breast cancer.^4^ Abemaciclib is another CDK4/6 inhibitor that is efficacious against ER+ and HER2− breast cancer when administered in combination with endocrine therapies, such as anti‐estrogen and aromatase inhibitors.^5^ It was approved in 2018.^4^, ^6^ The adverse effects of abemaciclib include myelosuppression, gastrointestinal symptoms, and skin reactions, which are clearly indicated on the package insert of abemaciclib.^7^ In addition, the Japanese Ministry of Health, Labour and Welfare has issued an alert regarding interstitial lung disease caused by abemaciclib.^8^ Similarly, for palbociclib, a directive has been provided to revise the package insert, and interstitial lung disease has been mentioned in the warning section.^9^ Acute pulmonary fibrosis, an interstitial lung disease, is fatal. Therefore, great care must be taken to prevent the development of this disease.^10^

However, considering that a large‐scale evaluation of the potential complication of interstitial lung disease has not been performed to date, its incidence is unclear. Here, we evaluated the incidence of interstitial lung disease associated with CDK4/6 inhibitor use in clinical practice. The Japanese Adverse Drug Event Report (JADER) is a database of voluntary reports of adverse events published by the Pharmaceuticals and Medical Devices Agency of Japan and is useful for the analysis of post‐marketing adverse event reports. In case of adverse events reported in the Food and Drug Administration Adverse Event Reporting System (FAERS) by the US Food and Drug Administration (FDA), signals of adverse events are detected using reported odds ratio (ROR).^11^ Using the data from these two databases, we assessed the association of palbociclib and abemaciclib with interstitial lung disease by detecting adverse event signals with ROR. In addition, we performed adverse event‐time analysis using data from the JADER database and examined the adverse reaction‐time profile of each drug.

## 2 METHODS

### 2.1 Databases

Data from the JADER database were downloaded from the website of the Pharmaceuticals and Medical Devices Agency (https://www.pmda.go.jp/) on 4 March 2020. The database consists of four files: demo, drug, reac, and hist. “Demo” presents basic patient information, such as sex, age, and weight. “Drug” presents the generic name of the drug, route of administration, and start and end dates of administration. “Reac” contains the name of adverse events, outcome, and date of onset of adverse events. “Hist” contains information about underlying diseases in patients.

Data from the FAERS were downloaded from the FDA website (http://www.fda.gov/) on 8 January 2020. The FAERS consists of seven files: DEMO, DRUG, REAC, OUTC, RPSR, INDI, and THER. “DEMO” consists of basic information, such as sex, age, date of adverse events, and the country where an adverse event occurred. “DRUG” contains information about drug names, route of administration, and dose information. “REAC” contains the name of adverse events and “OUTC” presents details about case outcomes. “RPSR” contains information on sources, such as the source of adverse events. Indications are presented in “INDI,” and the start and end dates of administration and treatment period are described in “THER.”

The analysis period for this study was from April 2004 to December 2019 when using the JADER database and from January 2004 to December 2019 when using the FAERS database. No ethical review is required for the use of FAERS and JADER data.^12^, ^13^ The Ethical Committee of Shujitsu University deemed that no review was necessary for this study.

### 2.2 Targets of analysis

The adverse event identified for extraction was interstitial lung disease as described in the Medical Dictionary for Regulatory Activities (MedDRA) Ver. 22.1J by the International Council of Harmonization (ICH), using standardized MedDRA queries. The basic term “interstitial lung disease” was set as the preferred term to narrow the scope terms. Abemaciclib and palbociclib were considered as the target drugs for analysis. In addition, sex and age were analyzed. Additional details on the reporting time of adverse reactions were also analyzed. Time‐onset analysis of drug administration and interstitial lung disease was performed only in cases where both the date of drug administration and date of onset of interstitial lung disease were clear. We used the free statistical software EZR (Easy R) to perform the log‐rank test.^14^

### 2.3 Analysis method

Access2016 (Microsoft) was used to create the JADER database. NaviCat for SQLite (Premium Soft) was used to create the FAERS. The safety evaluation of each anticancer drug was performed by signal detection. ROR was used as the index score^15^ and was calculated using a 2 × 2 contingency table that was categorized based on the use or non‐use of a drug and the presence or absence of specific adverse events. A signal was detected when the lower limit of a 95% confidence interval (CI) was greater than 1. In addition, signal detection for patients both over and under the age of 60 years was performed.^16^, ^17^, ^18^

The latest cases reported in the FAERS were retained, whereas the duplicated cases were excluded. Regarding the cases in the JADER database, cases with missing values (unclear age or sex) were excluded.

## 3 RESULTS

The total number of reports in the JADER database and that of reports on interstitial lung disease were 611 336 and 33 099, respectively. The total number of adverse events reported for abemaciclib and palbociclib was 151 and 1544, respectively, of which 38 and 50 were cases of interstitial lung disease. Abemaciclib or palbociclib were not administered to men. The RORs (95% CI) in the JADER for abemaciclib and palbociclib were 5.9 (95% CI: 4.1‐8.5) and 0.5 (95% CI: 0.4‐0.8), respectively. Among the two drugs, only abemaciclib had a lower limit of 95% CI of ROR greater than 1; thus, signals for only this drug were detected (Table 1). Interstitial lung disease has been reported for abemaciclib and palbociclib, at a mean of 65.1 days [median (interquartile range: 25th‐75th percentile): 56.0 days (25.3‐98.3)], and 53.1 days [38.0 (10.8‐76.0) days], respectively, from the start of treatment (Table 2). In the JADER database, regarding the association between the occurrence of interstitial lung disease and age, the ROR in patients under 60 years of age was 5.1 for abemaciclib (2.0‐13.0) and 0.5 for palbociclib (0.2‐1.3), and the ROR in patients over 60 years of age was 6.3 for abemaciclib (4.1‐9.5) and 0.8 for palbociclib (0.6‐1.2). The lower limit of the 95% CI of the ROR for abemaciclib was greater than 1, regardless of age, and a signal was detected (Table 3).

**TABLE 1:** Number of reports and the reporting odds ratio for each CDK4/6 inhibitor in the JADER database

Drug	Total number of adverse events	Number of cases of interstitial lung disease	ROR (95% CI)
Abemaciclib	151	38	5.9 (4.1–8.5)
Palbociclib	1544	50	0.6 (0.4–0.8)

Abbreviations: CDK 4/6, cyclin‐dependent kinase 4/6; CI, confidence interval; JADER, Japanese Adverse Drug Event Report; ROR, reported odds ratio.

**TABLE 2:** Median time‐to‐onset of interstitial lung disease reported in the Japanese Adverse Drug Event Report database

Drug	Number of reports	Average (day)	Median time‐to‐onset (day) (25%–75%)
Abemaciclib	18	65.1	56.0 (25.3–98.3)
Palbociclib	14	53.1	38.0 (10.8–76.0)

*Note*: The analysis was based on the data available on the reporting date and the date of drug administration.

**TABLE 3:** Age‐ and sex‐stratified analysis of CDK inhibitor‐related lung injury reported in the Japanese Adverse Drug Event Report database

JADER	Cases with interstitial lung disease	Cases with conditions other than interstitial lung disease	ROR (95% CI)
Age < 60 years			
Abemaciclib	5	33	5.1 (2.0–13.0)
Palbociclib	5	320	0.5 (0.2–1.3)
Age ≥ 60 years			
Abemaciclib	32	67	6.3 (4.1–9.5)
Palbociclib	35	557	0.8 (0.6–1.2)
Male			
Abemaciclib	0	0	0
Palbociclib	0	0	0
Female			
Abemaciclib	37	108	7.7 (5.3‐11.1)
Palbociclib	48	1148	0.9 (0.7‐1.2)

Abbreviations: CDK, cyclin‐dependent kinase; CI, confidence interval; JADER, Japanese Adverse Drug Event Report; ROR, reported odds ratio.

The total number of cases reported in the FAERS was 11 448 913, and the total number of reported adverse reactions related to interstitial lung disease was 66 335 (0.58%). The RORs (95% CI) for abemaciclib and palbociclib in the FAERS were 4.8 (95% CI: 3.8‐6.1) and 1.3 (95% CI: 1.1‐1.4), respectively. The lower limits of the 95% CI of the ROR for both drugs were greater than 1, and a signal was detected (Table 4). Regarding the association between the occurrence of interstitial lung disease and age, the ROR in patients under 60 years of age was 5.1 for abemaciclib (2.8‐9.2) and 1.4 for palbociclib (1.0‐1.8), and the ROR in patients over 60 years of age was 3.9 for abemaciclib (2.7‐5.4) and 0.6 for palbociclib (0.5‐0.7). The lower limit of the 95% CI of the ROR for abemaciclib was greater than 1, regardless of age, and a signal was detected (Table 5).

**TABLE 4:** Number of reports and the reporting odds ratio for each CDK inhibitor reported in the FAERS

Drug	Total	Case	ROR (95% CI)
Abemaciclib	2563	70	4.8 (3.8–6.1)
Palbociclib	37 496	276	1.3 (1.1–1.4)

Abbreviations: CDK, cyclin‐dependent kinase; CI, confidence interval; FAERS, FDA Adverse Event Reporting System; ROR, reported odds ratio.

**TABLE 5:** Age‐ and sex‐stratified analysis of CDK inhibitor‐related lung injury reported in the FDA Adverse Event Reporting System

FAERS	Cases with interstitial lung disease	Cases with conditions other than interstitial lung disease	ROR (95% CI)
Age < 60 years			
Abemaciclib	11	462	5.1 (2.8–9.2)
Palbociclib	58	9145	1.4 (1.0–1.8)
Age ≥ 60 years			
Abemaciclib	35	871	3.9 (2.7–5.4)
Palbociclib	133	20 207	0.6 (0.5–0.7)
Male			
Abemaciclib	0	0	0
Palbociclib	6	923	0.8 (0.4‐1.8)
Female			
Abemaciclib	65	2348	6.2 (4.8‐7.9)
Palbociclib	247	33 832	1.6 (1.4‐1.8)

Abbreviations: CDK, cyclin‐dependent kinase; CI, confidence interval; FAERS, FDA Adverse Event Reporting System; ROR, reported odds ratio.

## 4 DISCUSSION

Interstitial lung disease has been listed as a serious adverse effect in the package inserts of the CDK4/6 inhibitors abemaciclib and palbociclib.^7^, ^9^ Data of the analysis of reports from the JADER database showed that the lower limit of the 95% CI of the ROR for abemaciclib was greater than 1; however, the signal was not significant. In the analysis of reports from the FAERS, signals were detected for both abemaciclib and palbociclib. Individually, abemaciclib showed a stronger signal than palbociclib. Abemaciclib was more selective for CDK4 than palbociclib, and this may have contributed to the difference in the occurrence of adverse events.^19^ Nevertheless, the number of reports on palbociclib substantially in the FAERS exceeded that in the JADER database. Thus, it would be appropriate to conduct an analysis after an adequate number of cases have been reported in the JADER database.^11^

Furthermore, both abemaciclib and palbociclib were associated with the onset of interstitial lung disease after a delay of 1‐2 months from the start of treatment, indicating the need for monthly treatment monitoring in patients administered both drugs. In addition, the results showed that the common mechanism of action of these drugs may have contributed to the development of interstitial lung disease.

The results of this analysis showed that treatment with abemaciclib is associated with a possibility of development of interstitial lung disease, regardless of age. Although abemaciclib and palbociclib are commonly prescribed to patients older than 60 years, the association between age and the occurrence of CDK4/6 inhibitor‐induced interstitial lung disease needs to be further clarified owing to a smaller number of cases in Japan.

As abemaciclib and palbociclib are relatively new drugs in Japan (launched in November 2018 and December 2017, respectively), information on their adverse effects is limited. While palbociclib is a specific inhibitor of CDK4/6, abemaciclib can inhibit various kinases at normal doses; it inhibits the binding of cyclin B and CDK1 and has effects on the G1 and G2 phases of the cell cycle. In other words, abemaciclib has a low specificity for CDK4/6, and therefore, it may cause more severe adverse effects, which could explain the difference in the potential complication of interstitial pneumonia between abemaciclib and palbociclib.^20^ Hence, we were unable to perform an analysis based on disease severity. A sub‐analysis to explore the association of age and sex in the reports from the FAERS showed results similar to those from the JADER database (Table 5). This was a limitation of our study. In addition, although anticancer drugs are generally used in combination, we did not consider the combination of CDK4/6 inhibitors with other anticancer drugs in this analysis. Recently, the adjustment of confounding factors using logistic regression and subset analyses has been reported as an attempt to prove this point.^20^, ^21^, ^22^ The effects of concomitant medications and various confounding factors may be explored using such methods.^21^ The FAERS is suitable for screening unknown adverse events and analysis of trends in adverse events owing to the reports of large number of cases in this database. In contrast, the JADER database is useful for analyzing the trend of reporting domestic adverse events and its time of onset.^21^ Information on the onset time of adverse events is useful for alerting healthcare professionals and patients. Information on new agents, such as CDK4/6 inhibitors, is useful for maintaining patient quality of life in cases of adverse events, especially interstitial lung disease, wherein the mechanism of event onset is typically unknown, and no preventive or therapeutic measures have been established. In the analysis of the reports from the JADER database, only abemaciclib was found to have a signal >1 for the lower limit of the 95% CI for ROR. However, in the analysis of the reports from the FAERS, both abemaciclib and palbociclib were found to have a signal, which might be due to the racial differences between patients from Japan and the United States. Nevertheless, the number of cases in the JADER database was less than that in the FAERS.

As the ROR only shows a high frequency of adverse drug reaction reports, the causal relationship between the drugs and the adverse drug reactions has to be evaluated in the future. Furthermore, the adverse effect profiles of CDK4/6 inhibitors have not been systematically evaluated due to the small number of available cases. Nonetheless, we believe that this study provides important suggestions for future research.

In summary, the results of this study show that treatment with abemaciclib and palbociclib require caution owing to the potential complication of interstitial lung disease. We believe that these results may be useful in preventing the occurrence of interstitial lung disease when using CDK4/6 inhibitors. In addition, this method may be used to obtain information useful for the safety management of various other drugs.

## CONFLICT OF INTEREST

The authors declare no conflict of interest.

## AUTHOR CONTRIBUTIONS

**Hideki Nawa:** Conceptualization; data curation; formal analysis; funding acquisition; investigation; resources; visualization; writing‐original draft. **Takahiro Niimura:** Conceptualization; methodology; software; writing‐review & editing. **Kenta Yagi:** Validation; writing‐review & editing. **Mitsuhiro Goda:** Validation; writing‐review & editing. **Yoshito Zamami:** Supervision; writing‐review & editing. **Keisuke Ishizawa:** Project administration; writing‐review & editing.

## ETHICAL STATEMENT

No ethical review is required for the use of FAERS and JADER data. The Ethical Committee of Shujitsu University deemed that no review was necessary for this study.

## Data Availability

The datasets used and/or analyzed in this study are available from the corresponding author upon reasonable request.
